# Deriving analytical solutions using symbolic matrix structural analysis: Part 2 – Plane trusses

**DOI:** 10.1016/j.heliyon.2025.e42372

**Published:** 2025-02-06

**Authors:** Vagelis Plevris, Afaq Ahmad

**Affiliations:** a College of Engineering, Qatar University, P.O. Box: 2713, Doha, Qatar; b Department of Built Environment, Oslo Metropolitan University, P.O. Box 4, St. Olavs Plass, NO-0130, Oslo, Norway

**Keywords:** Matrix structural analysis, Symbolic analysis, Plane trusses, Structural analysis, Sensitivity analysis, Analytical solutions, Closed-form solutions

## Abstract

This study extends the use of symbolic computation in matrix structural analysis to plane trusses, expanding on previous work that focused on continuous beams. We present a fully open-source MATLAB program, available on GitHub, that performs symbolic analysis of 2D trusses subjected to point loads, applicable to any truss configuration. Using the Symbolic Math Toolbox, the program derives closed-form analytical expressions for displacements, support reactions, and axial forces, providing deeper insight into structural behavior. A key advantage of the symbolic approach is its ability to perform sensitivity analysis efficiently by computing partial derivatives of structural responses with respect to input parameters. This feature enhances design exploration and optimization by allowing direct evaluation of parameter influences. Moreover, the framework is highly scalable, capable of generating symbolic solutions even for large-scale truss structures, something previously unattainable using traditional methods due to computational limitations. The tool serves both engineering practice and education, offering clear insights into parameter relationships and strengthening conceptual understanding in structural mechanics. To ensure accuracy, the symbolic results were rigorously validated against two commercial finite element software programs and results from the literature, with complete agreement. These validations confirm the reliability, scalability, and general applicability of the proposed methodology.

## 1 Introduction

The Finite Element Method (FEM), which recently celebrated its 80th anniversary [1], is a cornerstone of structural analysis, providing a reliable numerical framework for solving complex engineering problems [2]. Traditional FEM, while effective for detailed results, has inherent limitations in flexibility and generality as numerical solutions are tied to specific inputs, requiring full re-computation for changes in material properties, geometry, or external loads [3]. These re-computations are time-consuming and computationally demanding, particularly for large-scale structures, and often obscure the relationships between key parameters, hindering a deeper understanding of structural behavior [4].

For linear structures like beams, trusses, and frames, FEM is often referred to as Matrix Structural Analysis (MatSA), a method that allows for the direct derivation of stiffness matrices without numerical integration. While MatSA simplifies the process and offers greater insight, it still depends heavily on predefined boundary conditions and loading scenarios. Any changes in the model or the loads require regenerating and recalculating the entire model, resulting in a repetitive and time-intensive workflow. This restricts adaptability in tasks like real-time analysis or mathematical optimization [5], where quick evaluation of multiple configurations is essential [6].

Before the advent of computers, most mathematical and engineering analyses relied heavily on closed formulas and symbolic computation, as numerical methods and solutions were virtually nonexistent. With the rise of computing technology, numerical methods quickly dominated scientific fields, offering efficient solutions to complex problems. However, efforts to bridge the gap between symbolic and numerical methods emerged, such as the development of the Macsyma system in the 1960s by MIT's AI group [7]. Symbolic computation can provide compelling solutions to challenges associated with numerical methods such as FEM and MatSA. Unlike purely numerical methods, which produce specific values, symbolic computation enables manipulation and solving of exact algebraic expressions, facilitating the derivation of analytical solutions and offering deeper insights into structural behavior.

MATLAB is widely used in structural engineering for its powerful numerical algorithms, addressing challenges related to matrix analysis of structures and FEM [[8], [9], [10], [11]], structural dynamics [[12], [13], [14], [15]], optimization [[16], [17], [18], [19], [20]], and others [21]. While renowned for numerical computing, MATLAB also supports symbolic computation through its Symbolic Math Toolbox [22,23]. The toolbox extends MATLAB's functionality, enabling symbolic algebraic simplifications, differentiation, integration, equation solving, and matrix manipulation [[24], [25], [26]]. For researchers and educators, it facilitates intuitive derivation of closed-form solutions and exploration of theoretical concepts, enhancing the understanding of complex systems. Its integration with MATLAB's numerical environment ensures seamless transitions between symbolic and numerical analyses, making it a versatile platform for both theoretical studies and practical applications. Other similar numerical platforms also support symbolic computation, like Mathematica [27], Maple [28], and SymPy (Python) [29].

Plane trusses often involve complex geometries with non-uniform node distribution and varying element lengths and orientations, which makes them less straightforward to model than linear systems like beams. The connections between multiple elements with varying orientations can lead to intricate force paths, requiring careful consideration in the analysis. Building on a previous work focused on continuous beams [4], this study extends the methodology to plane trusses, introducing capabilities specifically designed for 2D truss analysis. The study presents an innovative, open-source MATLAB program tailored for symbolic MatSA of 2D trusses subjected to joint point loads. For the first time, this program enables precise and efficient derivation of analytical solutions for any 2D truss configuration, offering symbolic expressions for node displacements, support reactions, and element axial forces. Key features of the program include.

• Fully open-source code, accessible online with comprehensive documentation and five illustrative numerical examples.
• Capability to derive closed-form solutions for various output quantities (e.g., displacements, support reactions, axial forces) for 2D trusses of any complexity.
• Support for sensitivity analysis, using MATLAB's built-in symbolic differentiation to assess the impact of input parameters on output quantities.
• Full validation of the symbolic results through comparison with established commercial finite element software and results from the literature, ensuring accuracy, reliability, and general applicability.

The source code, available on GitHub (https://github.com/vplevris/SymbolicMatSA-2DTrusses), includes all examples discussed in this study. Developed using MATLAB R2024b and its symbolic toolbox, the program is expected to be compatible with earlier versions. With clean, well-documented MATLAB code and minimal setup required, users are encouraged to explore the program, customize it to suit their specific needs, and efficiently generate their own analytical solutions. A preprint of this work can be found in Ref. [30].

## 2 Literature review

Truss structures, while relatively simple in form, hold significant importance in structural analysis due to their fundamental role in various engineering applications. As one of the earliest studied structural systems, trusses have historically provided a foundation for understanding load distribution and force transfer in structures. Despite being a well-established topic, the analysis of trusses remains a focus of continuous research, as engineers and researchers seek modern approaches to improve efficiency, accuracy, and scalability. For example, Parisi et al. [31] recently explored the use of mechanics-informed models and graph neural networks for structural analysis, demonstrating how advanced computational methods can enhance the modeling and understanding of truss behavior. This highlights how even fundamental topics in structural analysis, like trusses, are evolving with the adoption of cutting-edge technologies.

Few studies have addressed stiffness matrices symbolically. Eriksson and Pacoste [32] explored the use of symbolic software for developing finite element procedures, focusing on complex problems like higher-order instabilities requiring precise formulations. They emphasized that symbolic tools improve efficiency and clarity, enabling effective comparisons between element assumptions. Their research includes beam formulations for plane and space models, allowing analytical verification of equivalence between displacement and co-rotational approaches. Symbolic derivation also simplifies finite space rotations and systematically connects local displacements to global variables. Amberg et al. [33] developed a Maple-based toolbox for generating finite element codes, facilitating 1D, 2D, and 3D simulations. This toolbox has significantly accelerated research in areas like thermocapillary convection, welding, and crystal growth by reducing development time to hours. It offers flexibility, transparency, and ease of modification, enabling researchers to focus on physical insights while minimizing errors and debugging. Pavlović [34] highlighted symbolic computation as a powerful complement to traditional numerical methods in structural engineering. He reviewed its underutilized applications and emphasized its potential to advance classical structural analysis by addressing complex problems with greater efficiency. He advocated for integrating symbolic and numerical methods to leverage their complementary strengths in solving structural mechanics problems effectively.

Symbolic algebra has found diverse applications in structural engineering, enabling precise and insightful formulations of complex problems. For example, Levy et al. [35] utilized symbolic algebra to derive geometric stiffness matrices for membrane shells, allowing for the consideration of finite rotations without relying on small rotation assumptions. This approach enhances nonlinear analysis by providing explicit and physically intuitive derivations, demonstrating the power of symbolic methods in advancing structural mechanics. Murphey [36] derived generic symbolic equations for the effective stiffness and strength of beam-like trusses with arbitrary numbers of longerons and diagonal lacings, assuming relatively soft diagonals. These equations unify previous discrete cases and simplify truss design by allowing modifications through constant values. Covering bending, torsion, shear, and axial loading, the approach enables rapid preliminary sizing and optimization, validated against finite element analysis and prior results by Renton [37].

Skrinar and Pliberšek [38] derived a symbolic stiffness matrix and load vector for slender beams with transverse cracks under uniform loading. Using the principle of virtual work, they provided closed-form expressions that clarify the influence of crack depth and location on flexural deformation, aiding in crack identification and modeling per European design code EC8. Roque [39] explored symbolic and numerical analysis of bending plates using MATLAB, demonstrating its versatility in combining symbolic and numerical methods seamlessly to enhance problem-solving efficiency and accuracy.

Tinkov [40] derived new exact analytical expressions for the deflection of various planar trusses and analyzed existing solutions using the Maxwell-Mohr formula under elastic assumptions. Employing induction on the number of panels and symbolic computations in Maple, the study identified key characteristics and limitations related to panel count. Comparative analysis with known solutions, validated using the Lira software package, demonstrated the accuracy and applicability of the derived analytical expressions for truss deflection. Kirsanov and Tinkov [41] developed an algorithm to derive deflection and horizontal displacement formulas for planar statically determinate trusses under various loading conditions. Using symbolic mathematics in Maple, they generalized solutions for trusses with increasing panels, constructing and solving recurrence equations for polynomial coefficients. Their method identifies asymptotic properties and extreme points in deflection behavior, providing valuable benchmarks for validating numerical calculations in structural analysis. In a similar study, Kirsanov [42] proposed a rod model for planar statically determinate frames with four supports, deriving analytical deflection formulas under various loads using Maple. Through symbolic equilibrium equations, he identified kinematic instability for specific panel numbers and determined deflections for stable configurations using Maxwell-Mohr's formula. The method generalized solutions via double induction and recurrence equations, revealing significant deflection variations. Force expressions in critical rods were also derived, aiding design evaluation and optimization.

Dasgupta [43] explored the application of symbolic computations using Mathematica to derive equilibrium equations in terms of nodal displacements. Assuming linear elastic behavior and small displacements, the study demonstrates how bar stiffness matrices and nodal forces are integrated to construct the global system matrix. The Mathematica Solve function is employed to obtain exact solutions, offering an educational perspective on structural mechanics while showcasing the power of symbolic tools in finite element analysis. Öchsner and Makvandi [44] discuss the theory of single rod elements and plane truss structures, providing a detailed solution procedure through a comprehensive example. They combined manual calculations with insights into computer implementation using Maxima [45], enabling a clear understanding of the FEM. The work includes practical Maxima examples, facilitating the application of the methods to other problems and serving as a valuable resource for learning symbolic and numerical analysis in truss design.

In the field of structural optimization, truss structures remain a significant focus in the literature [[46], [47], [48]], despite their inherent simplicity. Analytical solutions continue to hold a distinct position in this domain, as exemplified by the work of Charalampakis and Chatzigiannelis [49]. Using the cylindrical algebraic decomposition (CAD) algorithm, they derived globally optimal solutions for minimum weight truss design. Their methodology, using symbolic computation, provides exact solutions to benchmark problems and serves as a formal validation of the convergence of metaheuristic methods to global optima.

## 3 Stiffness matrix of a plane truss element

The stiffness matrix is essential in MatSA as it establishes the relationship between applied forces and resulting displacements in a structural system. Typically, in MatSA and the FEM, stiffness matrices are computed numerically for each element and then assembled into a global system representing the entire structure. However, for linear elements in MatSA, it is often feasible to derive an exact symbolic expression for an element's stiffness matrix [44,50].

One such example is the 2D Truss Element, which has one degree of freedom (DOF) per node in the element's local system (displacement along the element's axis). This element is commonly used in linear static analysis of plane trusses. Fig. 1 shows the element in its local coordinate system, where the two DOFs—axial displacements a t each end—are illustrated. In plane trusses, each node of the model has two DOFs in the global coordinate system, corresponding to displacements in the *x* and *y* global directions. There are no rotations; each element experiences only axial deformation, resulting in axial forces and stresses, with no shear forces or bending moments.

**Fig. 1:**
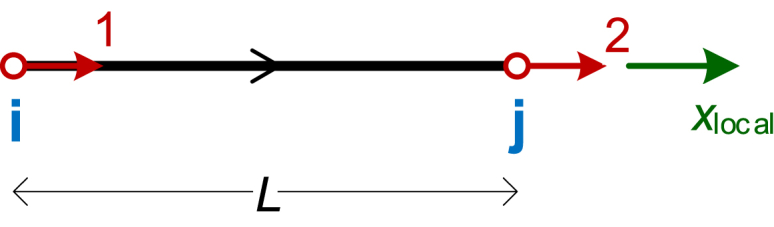
2D Euler–Bernoulli beam element with 6 DOFs.

Since plane truss elements can have various orientations on the plane, each element's stiffness matrix must be transformed (rotated) to a common coordinate system to construct the global stiffness matrix of the whole model. Fig. 2 shows an inclined element on the 2D plane, where the angle *θ* defines the rotation needed from the global *x*-axis to align with the local element axis with a counter-clockwise rotation. The figure also shows the local and global axes, with DOF numbering in the global system. For a 2D truss element, there are four DOFs in total, with two DOFs at each node, all referenced to the global axes, as shown in Fig. 2.

**Fig. 2:**
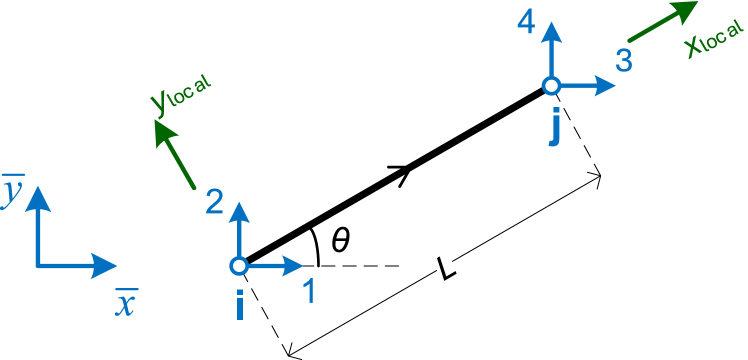
2D truss element with 4 DOFs in the global system.

The symbolic 2 × 2 stiffness matrix for the 2D truss element, corresponding to the two local DOFs depicted in Fig. 1, is expressed in Eq. (1).

(1) $$\left[{\hat{k}}_{t2}\right]=\frac{EA}{L}\cdot \left[\begin{array}{cc} 1 & -1\\ -1 & 1 \end{array}\right]$$

The transformation matrix, which is a 2 × 4 matrix, is given by Eq. (2) [44].

(2) $$\left[T_{t2}\right]=\left[\begin{array}{cccc} c & s & 0 & 0\\ 0 & 0 & c & s \end{array}\right]$$

Where *c* and *s* are given by Eqs. (3), (4):

(3) $$c=\cos\left(\theta \right)=\frac{\Delta x}{L}=\frac{x_{j}-x_{i}}{L}$$

(4) $$s=\sin\left(\theta \right)=\frac{\Delta y}{L}=\frac{y_{j}-y_{i}}{L}$$

The global stiffness matrix $\left[{\bar{k}}_{t2}\right]$ of the element, a 4 × 4 matrix corresponding to the four DOFs illustrated in Fig. 2, is obtained using the expression of Eq. (5) [44]:

(5) $$\left[{\bar{k}}_{t2}\right]={\left[T_{t2}\right]}^{T}\cdot \left[{\hat{k}}_{t2}\right]\cdot \left[T_{t2}\right]=\frac{EA}{L}\cdot \left[\begin{array}{cccc} c^{2} & cs & -c^{2} & -cs\\ cs & s^{2} & -cs & -s^{2}\\ -c^{2} & -cs & c^{2} & cs\\ -cs & -s^{2} & cs & s^{2} \end{array}\right]$$

In these equations, *E* is Young's modulus of the material, *A* is the cross-sectional area, and *L* is the length of the truss element.

## 4 Definition of the model symbolically

In Part 1 of this work [4], which focused on continuous beams, the beam model was defined symbolically using variables such as *Lengths*, Supports, *PointLoads*, and *UniformLoads*. This approach was suitable for beams because all elements were aligned along a single axis, making it straightforward to describe the structure using only the lengths of elements and continuous numbering for nodes and elements. In the case of 2D trusses, however, the geometry is more complex. Nodes can connect multiple elements at various orientations, and the structure requires a more general representation. For this reason, we have introduced a set of variables that can effectively describe any 2D truss configuration.

• *NodeCoords*: A matrix of size *NumNodes* × 2, where each row defines the *x*- and *y*-coordinates of a node. The first column corresponds to the *x*-coordinate, and the second column corresponds to the *y*-coordinate. The values can be either numeric or symbolic (e.g. *L*).
• *ElemMatSec*: A column vector of size *NumElements*, where each entry represents the stiffness property of an element, defined as the product *E*⋅*A.* Here, *E* is the modulus of elasticity, and *A* is the cross-sectional area of the element. The entries can be numeric or symbolic (e.g. *EA*).
• *ElemCon*: A matrix of size *NumElements* × 2, where each row specifies the connectivity of an element by defining the IDs of its start and end nodes. The first column corresponds to the start node, and the second column corresponds to the end node. Each Node ID has a numeric value.
• *Supports*: A matrix of size *NumNodes* × 2, where each row defines the boundary conditions at a node. The first column corresponds to the *x*-direction, and the second column corresponds to the *y*-direction. A value of 1 indicates the degree of freedom (DOF) is constrained (fixed), while 0 indicates it is free.
• *PointLoads*: A matrix of size *NumNodes* × 2, where each row defines the external loads applied to a node. The first column specifies the force in the *x*-direction, and the second column specifies the force in the *y*-direction. The loads can be numeric or symbolic (e.g. *P*).

This general input file format provides a robust framework for describing any 2D truss configuration. By defining the coordinates of nodes and the connectivity of elements, the program automatically calculates element lengths and orientations, eliminating the need for manual computation of geometric properties. This flexibility allows the analysis of intricate truss geometries and ensures scalability for large structures.

The symbolic approach also supports input parameters that can be defined either as fixed numeric values or as symbolic variables. This enables advanced applications such as parameterized studies, sensitivity analysis, and design optimization. The generality of this format ensures compatibility with a wide range of truss problems, providing a versatile and efficient framework for 2D truss analysis. The general format of an input file is as follows:

**Figure:**
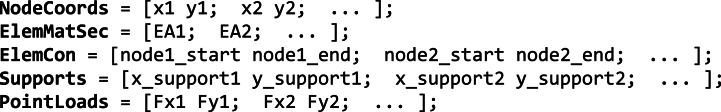

It is important to emphasize that an input file for the program does not define a single, specific truss structure but rather represents a family of truss structures described using symbolic parameters. Additionally, due to the symbolic nature of the tool, the results are presented as mathematical expressions instead of graphical representations. To visualize or further analyze specific truss configurations, users must substitute numeric values into the symbolic solutions. This process is demonstrated later in the manuscript in the section detailing the validation of the results.

## 5 Importance and educational benefits of symbolic solutions in 2D truss analysis

Symbolic representation in structural analysis provides engineers and researchers with valuable insights into structural behavior. By preserving algebraic relationships between parameters, symbolic MatSA enables detailed exploration of how variations in material properties, geometry, or loading conditions influence the overall structural response. This approach offers parameterized solutions that are not limited to specific input values, providing unmatched flexibility and adaptability. Additionally, symbolic expressions allow for straightforward calculation of partial derivatives with respect to input parameters, making sensitivity analysis and design optimization more efficient and accessible.

Symbolic MatSA holds significant educational value. It enhances the understanding of structural engineering concepts by clearly demonstrating the relationships between key parameters. Unlike purely numerical methods, which deliver results without revealing the underlying mechanics, symbolic solutions offer a transparent view of these interactions, fostering a deeper conceptual understanding. For example, consider a 2D truss element, like the one of Fig. 1, which is pinned at one end (no displacement) and is subjected to an axial load *P* at the other (free) end. Using symbolic MatSA, or even classical mechanics for this simple case, the displacement at that free node can be expressed as shown in Eq. (6):

(6) $$D_{x}=\frac{PL}{AE}$$

where *P* is the applied axial load, *L* is the truss member length, *A* is the cross-sectional area, and *E* is Young's modulus. This equation explicitly shows the direct proportionality of displacement to the applied load and member length, and the inverse proportionality to material stiffness and cross-sectional area. Such relationships are not apparent in a purely numerical solution, which provides only a single numerical result for given input values. Students can clearly see how increasing *L* or reducing *A* affects the displacement, fostering better intuition for design decisions.

Symbolic solutions also aid in understanding the concept of superposition and the cumulative effect of different loads on a truss system. By analyzing symbolic expressions for axial forces or nodal displacements, students can observe how various load cases combine to contribute to the overall response. This level of clarity may not be achievable through numerical results alone, where the contributions of individual load cases are often obscured.

Another significant advantage lies in sensitivity analysis and optimization. Symbolic expressions allow direct differentiation with respect to input parameters such as *E* or *A*, enabling students to explore how changes in material or geometric properties impact truss performance. For instance, differentiating the displacement equation of Eq. (6) with respect to *A* shows the rate of change of displacement due to variations in cross-sectional area, highlighting the sensitivity of the structure to design changes, as shown in Eq. (7):

(7) $$\frac{\partial D_{x}}{\partial A}=-\frac{PL}{A^{2}E}$$

This expression shows that the rate of change of displacement with respect to the cross-sectional area *A* is inversely proportional to *A*^2^. As *A* increases, the displacement decreases at a diminishing rate, illustrating the sensitivity of the structure's displacement to changes in its cross-sectional area. Sensitivity analysis and partial derivatives are examined in more detail in Section 8 of the manuscript.

In general, symbolic analysis provides a transparent and accessible way to understand the relationships between structural parameters and the response of truss systems. By exposing these relationships, symbolic MatSA enhances learning, equips students with the skills needed for parametric studies, and offers a robust foundation for sensitivity analysis and optimization. This approach serves as both an effective educational tool and a practical link between theoretical concepts and real-world applications in structural engineering.

## 6 Numerical examples

We consider five numerical examples of differing levels of complexity. In these, *EA* is treated as a single symbolic parameter, since *E* and *A* consistently appear together in stiffness and other analytical expressions in 2D trusses. Nevertheless, each member can have its own *EA* properties, i.e. a member can be stiffer than another. Table 1 provides detailed descriptions of the numerical examples and the associated symbolic parameters for each. The input files of all numerical examples can be found in the Appendix of this work.

**Table 1:** Details of the five numerical examples.

**Example #**	**Figure**		**Symbolic Parameters**
1			3 (*EA*, *L*, and *P*)
2 and 3			4 (*EA*, *L*, *H*, and *P*)
4			4 (*EA*, *L*, *H*, and *P*)
5			4 (*EA*, *L*, *H*, and *P*)

### 6.1 Numerical example 1 (3 symbolic parameters)

The first numerical example is a simple 2-bar truss with two members and a point load *P*, as shown in Fig. 3. The symbolic parameters are three: *EA*, *L*, and *P*.

**Fig. 3:**
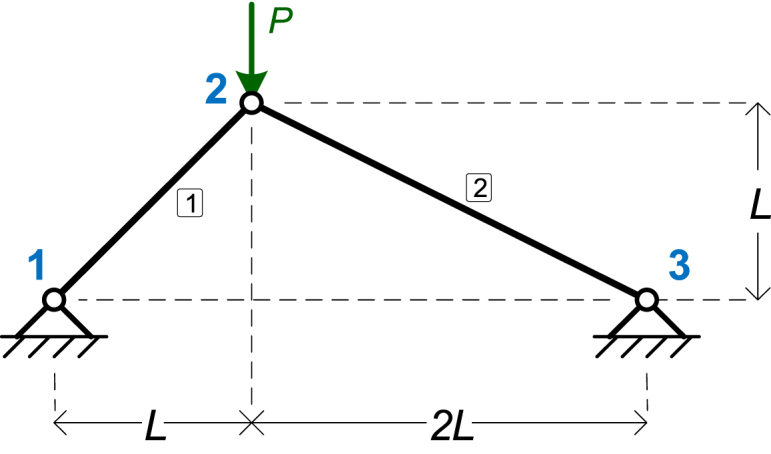
The truss of Example 1.

Table 2 shows the details of the model, as given in MATLAB. In every example, the number of nodes (*NumNodes*) is defined by the rows of the *NodeCoords* matrix and the number of elements (*NumElements*) is defined by the rows of the *ElemMatSec* matrix. In this example we have 2 elements and 3 nodes. In a 2D truss, all point loads are defined on nodes. There are no uniform loads.

**Table 2:** Details of the input parameters of the 1st numerical example.

**NodeCoords**	${\left[\begin{array}{ccc} 0 & L & 3L\\ 0 & L & 0 \end{array}\right]}^{T}$
**ElemMatSec**	${\left[\begin{array}{cc} EA & EA \end{array}\right]}^{T}$
**ElemCon**	${\left[\begin{array}{cc} 1 & 2\\ 2 & 3 \end{array}\right]}^{T}$
**Supports**	${\left[\begin{array}{ccc} 1 & 0 & 1\\ 1 & 0 & 1 \end{array}\right]}^{T}$
**PointLoads**	${\left[\begin{array}{ccc} 0 & 0 & 0\\ 0 & -P & 0 \end{array}\right]}^{T}$

Table 3, Table 4, Table 5 present the results of the symbolic analysis in terms of the symbolic parameters. Table 3 presents the Node displacements, while Table 4 shows the support reactions and Table 5 the element axial forces. The element forces are reported here as positive when the member is in tension and negative when the member is under compression. The analytical expressions for the element stresses are not reported in the results, as the stress for any element can be easily found by dividing the force of the element with its cross-sectional area, *A*.

**Table 3:** Example 1: Node displacements.

Node #	*x*-Displacement (*D*_*x*_)	*y*-Displacement (*D*_*y*_)
Node 1	0	0
Node 2	$-\frac{PL\left(4\sqrt{2}-5\sqrt{5}\right)}{9EA}$	$-\frac{PL\left(8\sqrt{2}+5\sqrt{5}\right)}{9EA}$
Node 3	0	0

**Table 4:** Example 1: Support reactions.

Node #	Force *F*_*x*_	Force *F*_*y*_
Node 1	2P/3	2P/3
Node 3	−2P/3	P/3

**Table 5:** Example 1: Element axial forces.

Element #	Axial Force
1	$-\frac{2\sqrt{2}}{3}P$
2	$-\frac{\sqrt{5}}{3}P$

### 6.2 Numerical example 2 (4 symbolic parameters)

The second numerical example is the truss shown in Fig. 4. It has 3 nodes and 3 elements. The symbolic parameters are four: *EA*, *L*, *H*, and *P*.

**Fig. 4:**
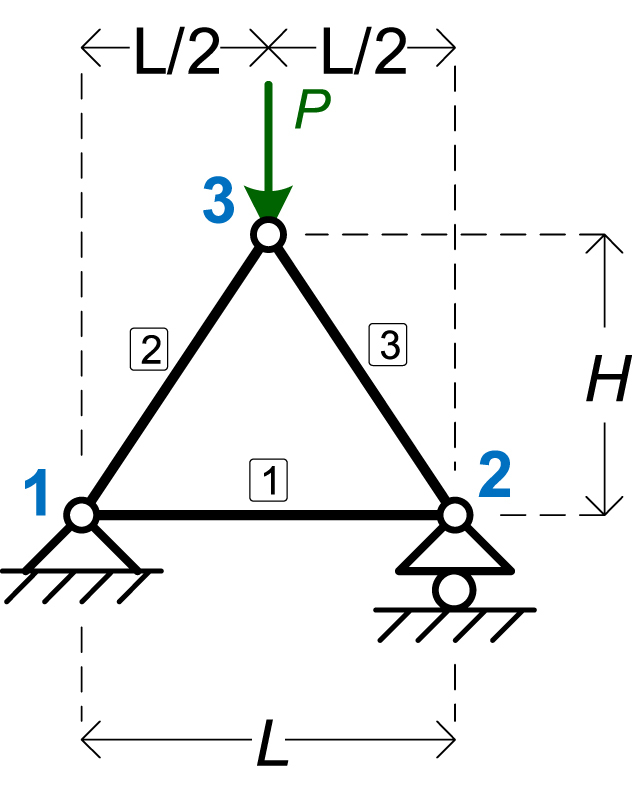
The truss of Example 2.

Table 6 shows the details of the model, as given in MATLAB.

**Table 6:** Details of the input parameters of the 2nd numerical example.

**NodeCoords**	${\left[\begin{array}{ccc} 0 & L & L/2\\ 0 & 0 & H \end{array}\right]}^{T}$
**ElemMatSec**	${\left[\begin{array}{ccc} EA & EA & EA \end{array}\right]}^{T}$
**ElemCon**	${\left[\begin{array}{ccc} 1 & 1 & 2\\ 2 & 3 & 3 \end{array}\right]}^{T}$
**Supports**	${\left[\begin{array}{ccc} 1 & 0 & 0\\ 1 & 1 & 0 \end{array}\right]}^{T}$
**PointLoads**	${\left[\begin{array}{ccc} 0 & 0 & 0\\ 0 & 0 & -P \end{array}\right]}^{T}$

The results of the symbolic analysis are given in Table 7, Table 8, Table 9, for Node displacements, support reactions, and element axial forces, respectively.

**Table 7:** Example 2: Node displacements.

Node #	*x*-Displacement (*D*_*x*_)	*y*-Displacement (*D*_*y*_)
Node 1	0	0
Node 2	$\frac{PL^{2}}{4EAH}$	0
Node 3	$\frac{PL^{2}}{8EAH}$	$-\frac{P\left({\left(4H^{2}+L^{2}\right)}^{3/2}+L^{3}\right)}{16EAH^{2}}$

**Table 8:** Example 2: Support reactions.

Node #	Force *F*_*x*_	Force *F*_*y*_
Node 1	0	$\frac{P}{2}$
Node 3	–	$\frac{P}{2}$

**Table 9:** Example 2: Element axial forces.

Element #	Axial Force
1	$\frac{PL}{4H}$
2	$-\frac{P\sqrt{4H^{2}+L^{2}}}{4H}$
3	$-\frac{P\sqrt{4H^{2}+L^{2}}}{4H}$

### 6.3 Numerical example 3 (4 symbolic parameters)

The third numerical example is the truss shown in Fig. 5. It has 5 nodes and 7 elements. The symbolic parameters are four: *EA*, *L*, *H*, and *P*.

**Fig. 5:**
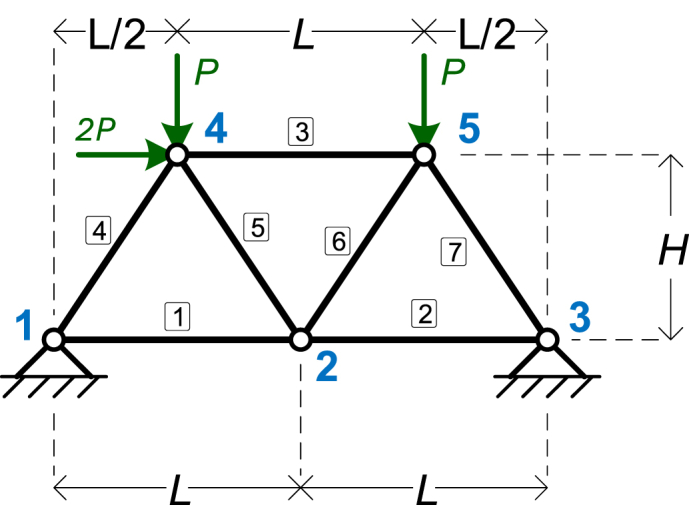
The truss of Example 3.

Table 10 shows the details of the model, as given in MATLAB.

**Table 10:** Details of the input parameters of the 3rd numerical example.

**NodeCoords**	${\left[\begin{array}{ccccc} 0 & L & 2L & L/2 & 3L/2\\ 0 & 0 & 0 & H & H \end{array}\right]}^{T}$
**ElemMatSec**	${\left[\begin{array}{ccccccc} EA & EA & EA & EA & EA & EA & EA \end{array}\right]}^{T}$
**ElemCon**	${\left[\begin{array}{ccccccc} 1 & 2 & 4 & 1 & 2 & 2 & 3\\ 2 & 3 & 5 & 4 & 4 & 5 & 5 \end{array}\right]}^{T}$
**Supports**	${\left[\begin{array}{ccccc} 1 & 0 & 1 & 0 & 0\\ 1 & 0 & 1 & 0 & 0 \end{array}\right]}^{T}$
**PointLoads**	${\left[\begin{array}{ccccc} 0 & 0 & 0 & 2P & 0\\ 0 & 0 & 0 & -P & -P \end{array}\right]}^{T}$

The results of the symbolic analysis are given in Table 11, Table 12, Table 13, for Node displacements, support reactions, and element axial forces, respectively.

**Table 11:** Example 3: Node displacements.

Node #	*x*-Displacement (*D*_*x*_)	*y*-Displacement (*D*_*y*_)
Node 1	0	0
Node 2	$\frac{PL}{2EA}$	$-\frac{P\left(2HL^{2}+\frac{{\left(4H^{2}+L^{2}\right)}^{3/2}}{2}+L^{3}\right)}{4EAH^{2}}$
Node 3	0	0
Node 4	$\frac{P\left(H{\left(4H^{2}+L^{2}\right)}^{3/2}+3HL^{3}+L^{4}\right)}{4EAHL^{2}}$	$-\frac{P\left(3HL^{2}+{\left(4H^{2}+L^{2}\right)}^{3/2}+L^{3}\right)}{8EAH^{2}}$
Node 5	$-\frac{P\left(HL^{3}-H{\left(4H^{2}+L^{2}\right)}^{3/2}+L^{4}\right)}{4EAHL^{2}}$	$-\frac{P\left(HL^{2}+{\left(4H^{2}+L^{2}\right)}^{3/2}+L^{3}\right)}{8EAH^{2}}$

**Table 12:** Example 3: Support reactions.

Node #	Force *F*_*x*_	Force *F*_*y*_
Node 1	$-\frac{P\left(2H-L\right)}{2Η}$	$-\frac{P\left(H-L\right)}{L}$
Node 3	$-\frac{P\left(2H+L\right)}{2H}$	$\frac{P\left(H+L\right)}{L}$

**Table 13:** Example 3: Element axial forces.

Element #	Axial Force
1	$\frac{P}{2}$
2	$-\frac{P}{2}$
3	$-\frac{P\left(2H+L\right)}{2H}$
4	$\frac{P\sqrt{4H^{2}+L^{2}}\cdot \left(H-L\right)}{2HL}$
5	$-\frac{P\sqrt{4H^{2}+L^{2}}}{2L}$
6	$\frac{P\sqrt{4H^{2}+L^{2}}}{2L}$
7	$-\frac{P\sqrt{4H^{2}+L^{2}}\cdot \left(H+L\right)}{2HL}$

### 6.4 Numerical example 4 (4 symbolic parameters)

The fourth numerical example is the truss structure shown in Fig. 6. The symbolic parameters are four: *EA*, *L*, *H*, and *P*.

**Fig. 6:**
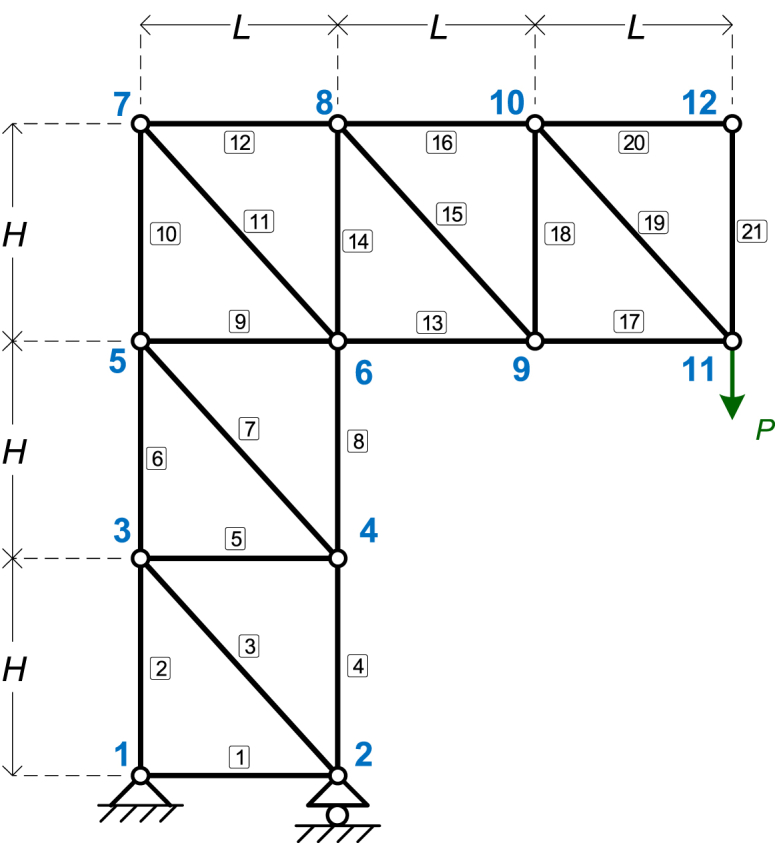
The truss of Example 4.

This model is relatively large, comprising 12 nodes and 21 elements. Detailed specifications of the model can be found in the input file for Example 4 in the source code repository. Each element is defined sequentially from the lower-numbered node to the higher-numbered node; for instance, element 7 connects nodes 4 and 5 in that order, while element 15 connects nodes 8 and 9. The supports include a pinned support at node 1 and a roller support at node 2, as illustrated in the figure.

The results of the symbolic analysis are reported in Table 14, Table 15, Table 16, for Node displacements (for selected nodes); support reactions; and element axial forces (for all elements), respectively.

**Table 14:** Example 4: Node displacements (for selected nodes).

Node #	*x*-Displacement (*D*_*x*_)	*y*-Displacement (*D*_*y*_)
Node 6	$\frac{9H^{2}P}{AEL}$	$-\frac{6HP}{AE}$
Node 7	$\frac{P\left(2{\left(H^{2}+L^{2}\right)}^{3/2}+21H^{3}\right)}{EAHL}$	$\frac{6HP}{AE}$
Node 9	$\frac{P\left(9H^{3}-2L^{3}\right)}{EAHL}$	$-\frac{P\left(3{\left(H^{2}+L^{2}\right)}^{3/2}+19H^{3}+4L^{3}\right)}{EAH^{2}}$
Node 11	$\frac{3P\left(3H^{3}-L^{3}\right)}{EAHL}$	$-\frac{2P\left(3{\left(H^{2}+L^{2}\right)}^{3/2}+16H^{3}+5L^{3}\right)}{EAH^{2}}$

**Table 15:** Example 4: Support reactions.

Node #	Force *F*_*x*_	Force *F*_*y*_
Node 1	0	−2P
Node 2	–	3P

**Table 16:** Example 4: Element axial forces.

Element #	Axial Force	Element #	Axial Force
1	0	11	$-\frac{2P\sqrt{H^{2}+L^{2}}}{H}$
2	2P	12	$\frac{2LP}{H}$
3	0	13	$-\frac{2LP}{H}$
4	−3P	14	−P
5	0	15	$\frac{P\sqrt{H^{2}+L^{2}}}{H}$
6	2P	16	$\frac{LP}{H}$
7	0	17	$-\frac{LP}{H}$
8	−3P	18	−P
9	0	19	$\frac{P\sqrt{H^{2}+L^{2}}}{H}$
10	2P	20	0
		21	0

### 6.5 Numerical example 5 (4 symbolic parameters)

The fifth numerical example is taken from the literature. In particular, it is the case No 1 truss model from the work of Tinkov [40]. The truss is shown in Fig. 7. The symbolic parameters are four: *EA*, *L*, *H*, and *P*.

**Fig. 7:**
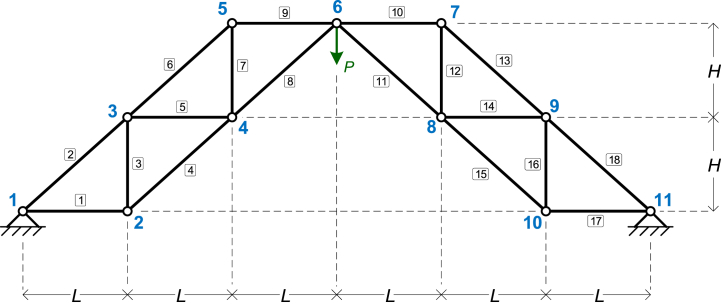
The truss of Example 5.

The model consists of 11 nodes and 18 elements, with detailed specifications available in the input file for Example 5 in the source code repository. Each element is defined sequentially, connecting the lower-numbered node to the higher-numbered node. The supports are pinned at nodes 1 and 11, as shown in the figure. The model is fully symmetric, including its loading, so identical results are expected on both sides of the truss.

The results of the symbolic analysis are given in Table 17, Table 18, Table 19, for Node displacements (for selected nodes); support reactions; and element axial forces, respectively.

**Table 17:** Example 5: Node displacements (for selected nodes).

Node #	*x*-Displacement (*D*_*x*_)	*y*-Displacement (*D*_*y*_)
Node 2	$-\frac{L^{2}P}{4EAH}$	$-\frac{P\left(4{\left(H^{2}+L^{2}\right)}^{3/2}+2H^{3}+L^{3}\right)}{8EAH^{2}}$
Node 4	$-\frac{L^{2}P}{8EAH}$	$-\frac{P\left(3{\left(H^{2}+L^{2}\right)}^{3/2}+H^{3}+L^{3}\right)}{4EAH^{2}}$
Node 6	0	$-\frac{P\left(10{\left(H^{2}+L^{2}\right)}^{3/2}+2H^{3}+3L^{3}\right)}{8EAH^{2}}$
Node 8	$\frac{L^{2}P}{8EAH}$	$-\frac{P\left(3{\left(H^{2}+L^{2}\right)}^{3/2}+H^{3}+L^{3}\right)}{4EAH^{2}}$
Node 10	$\frac{L^{2}P}{4EAH}$	$-\frac{P\left(4{\left(H^{2}+L^{2}\right)}^{3/2}+2H^{3}+L^{3}\right)}{8EAH^{2}}$

**Table 18:** Example 5: Support reactions.

Node #	Force *F*_*x*_	Force *F*_*y*_
Node 1	$\frac{3LP}{4H}$	$\frac{P}{2}$
Node 11	$-\frac{3LP}{4H}$	$\frac{P}{2}$

**Table 19:** Example 5: Element axial forces.

Element #	Axial Force	Element #	Axial Force
1	$-\frac{PL}{4H}$	10	$-\frac{LP}{4H}$
2	$-\frac{P\sqrt{H^{2}+L^{2}}}{2H}$	11	$-\frac{P\sqrt{H^{2}+L^{2}}}{2H}$
3	$\frac{P}{4}$	12	$\frac{P}{4}$
4	$-\frac{P\sqrt{H^{2}+L^{2}}}{4H}$	13	$-\frac{P\sqrt{H^{2}+L^{2}}}{4H}$
5	$-\frac{LP}{4H}$	14	$-\frac{LP}{4H}$
6	$-\frac{P\sqrt{H^{2}+L^{2}}}{4H}$	15	$-\frac{P\sqrt{H^{2}+L^{2}}}{4H}$
7	$\frac{P}{4}$	16	$\frac{P}{4}$
8	$-\frac{P\sqrt{H^{2}+L^{2}}}{2H}$	17	$-\frac{LP}{4H}$
9	$-\frac{LP}{4H}$	18	$-\frac{P\sqrt{H^{2}+L^{2}}}{2H}$

## 7 Validation of results

The validation of any result is a critical step in establishing its reliability, especially for symbolic solutions, which are designed to be general and applicable to various configurations. Unlike numerical methods that directly produce specific results for a given set of inputs, symbolic solutions retain algebraic relationships, making them more versatile but also requiring rigorous validation to ensure accuracy across diverse scenarios. Validation is essential to confirm that the derived symbolic expressions accurately represent the behavior of the structural system for any configuration.

A systematic validation procedure was employed in this study. Using MATLAB's *subs* command, all symbolic variables were replaced with numeric values, allowing the symbolic results to be expressed numerically for direct comparison with other finite element software. This enabled precise evaluation of the symbolic results for specific truss configurations. Two software packages were utilized for this comparison: SAP2000 Ultimate (v21.2) and EngiLab Truss.2D 2022 Pro (v1.3). Additionally, for one example, comparisons were made with closed-form solutions from the literature.

### 7.1 Validation with SAP2000

All five truss examples were validated against SAP2000, with results matching perfectly for node displacements, support reactions, and element axial forces. Here, we present the validation for the third example, illustrated in Fig. 5. The following properties were used for numerical calculations.

• Length (*L*): 8 m
• Height (*H*): 6 m
• Axial stiffness (*EA*): Derived using *E* = 200 × 10^6^ kN/m^2^ (200 GPa) and *A* = 4 × 10^−4^ m^2^ (4 cm^2^), resulting in *EA* = 8 × 10^4^ kN.
• Point load (*P*): 100 kN

**Fig. 8:**
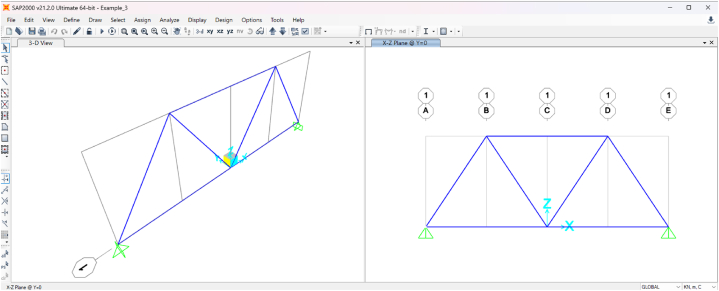
The truss model of Example 3, modeled with SAP2000.

The truss was modeled in SAP2000, as depicted in Fig. 8.

Table 20 presents a comparison of element axial forces between the symbolic software and SAP2000. The results are identical, apart from minor differences in decimals due to rounding. This consistency extends to node displacements and support reactions, though those results are not shown here.

**Table 20:** Element axial forces for Example 3 – Comparison with SAP2000.

Element #	Axial Force	Numeric value of symbolic expression	SAP2000 Result
1	$\frac{P}{2}$	50	50
2	$-\frac{P}{2}$	−50	−50
3	$-\frac{P\left(2H+L\right)}{2H}$	−166.667	−166.666
4	$\frac{P\sqrt{4H^{2}+L^{2}}\cdot \left(H-L\right)}{2HL}$	−30.0463	−30.046
5	$-\frac{P\sqrt{4H^{2}+L^{2}}}{2L}$	−90.1388	90.138
6	$\frac{P\sqrt{4H^{2}+L^{2}}}{2L}$	90.13878	−90.139
7	$-\frac{P\sqrt{4H^{2}+L^{2}}\cdot \left(H+L\right)}{2HL}$	−210.324	−210.323

It is important to note that SAP2000 includes shear deformations by default in all frame and truss models. However, the Euler–Bernoulli beam theory, employed in our program, neglects the effects of shear deformation, as it assumes that transverse shear strain is insignificant. To ensure a meaningful comparison between the results from our symbolic program and SAP2000, the shear effects in SAP2000 were effectively minimized by setting the frame property modifier for shear area to a very high value (10^6^). This adjustment aligns the SAP2000 model with the assumptions of the Euler–Bernoulli theory, allowing for consistent and accurate validation of our results.

### 7.2 Validation with EngiLab truss.2D pro

All five truss examples were also validated using EngiLab Truss.2D 2022 Pro, a specialized software for plane truss analysis. In every case, the results for node displacements, support reactions, and element axial forces matched perfectly. The validation for the fourth example, shown in Fig. 6, is presented here. The following properties were used for numerical calculations.

• Length (*L*): 5 m
• Height (*H*): 6 m
• Axial stiffness (*EA*): Derived using *E* = 200 × 10^6^ kN/m^2^ (200 GPa) and *A* = 2 × 10^−3^ m^2^ (20 cm^2^), resulting in *EA* = 4 × 10^5^ kN.
• Point load (*P*): 50 kN

**Fig. 9:**
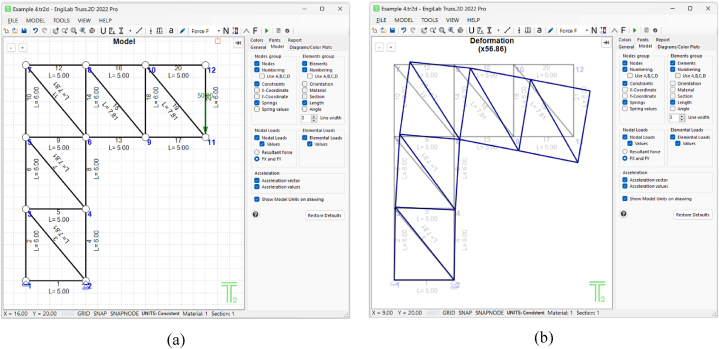
The truss of Example 4, modeled with EngiLab Truss.2D Pro: (a) Truss model, (b) Deformed state of the model.

The truss was modeled in EngiLab Truss.2D Pro, as illustrated in Fig. 9 where the truss model is shown in Fig. 9(a), together with its deformed shape in Fig. 9(b).

Table 21 compares node displacements between our symbolic software and EngiLab Truss.2D Pro, showing identical results. This consistency also applies to support reactions and element axial forces, though these results are not included here.

**Table 21:** Node displacements for Example 3 (for selected nodes) – Comparison with EngiLab Truss.2D Pro.

	Numeric value of symbolic expression		EngiLab Truss.2D Pro Result	
Node #	*x*-Displacement (*D*_*x*_)	*y*-Displacement (*D*_*y*_)	*x*-Displacement (*D*_*x*_)	*y*-Displacement (*D*_*y*_)
Node 6	0.0081	−0.0045	0.0081	−0.0045
Node 7	0.02287	0.0045	0.02287	0.0045
Node 9	0.007058	−0.02095	0.00706	−0.02095
Node 11	0.006538	−0.03827	0.00654	−0.03827

### 7.3 Validation with results from the literature

The fifth truss example is based on a case from the literature, specifically Case No. 1 from the work of Tinkov [40]. In this study, the author presents an analytical formula for calculating the vertical displacement of the central node (node 6 in our model). The formula is the one shown in Eq. (8):

(8) $$\Delta_{6}=\frac{P}{EF}\cdot \frac{Aa^{3}+Bb^{3}+Cc^{3}}{Db^{2}}$$

Tinkov's methodology provides analytical solutions for displacements in various truss configurations, using the parameter *n*, which defines the truss topology and the number of repetitions of the main truss pattern, for each truss configuration. For the specific truss model shown in Fig. 7, it is *n* = 2. According to the study [40], the values of the parameters *A*, *B*, *C*, and *D* for the specific truss are as follows.

• *A* = *n*+1 = 3
• *B* = *n* = 2
• $C=\frac{n\left(n+1\right)\left(2n+1\right)}{3}=10$
• *D* = 2*n*^2^ = 8
• *a* = *L* (the *L* symbolic variable used in our study)
• *b* = *H* (the *H* symbolic variable used in our study)
• $c=\sqrt{a^{2}+b^{2}}=\sqrt{L^{2}+H^{2}}$

It is important to note that in Eq. (8) the variable *F* represents the cross-sectional area. Substituting *F* with our variable *A* for the cross-sectional area, and taking the above values of the parameters into account, the final formula for the vertical displacement of node 6, as derived in Ref. [40], is the one of Eq. (9):

(9) $$\Delta_{6}=\frac{P}{EA}\cdot \frac{3L^{3}+2H^{3}+10{\left(L^{2}+H^{2}\right)}^{3/2}}{8H^{2}}$$

Eq. (9) fully matches the formula derived by our program for the vertical displacement of node 6, as presented in Table 17. The negative sign in the equation from Table 17 indicates that the displacement is directed downwards.

## 8 Sensitivity analysis

Sensitivity analysis is an essential tool in structural engineering for evaluating how variations in input parameters influence a system's response. It is critical for assessing the robustness of a design and optimizing performance by identifying the parameters that most significantly impact structural behavior. Engineers often seek to understand how changes in material properties, geometric dimensions, or applied loads affect displacements, stresses, reaction forces, or internal forces. By offering insights into these relationships, sensitivity analysis facilitates more informed decision-making during the design process.

Symbolic solutions provide a distinct advantage for conducting sensitivity analysis. With symbolic representations of structural behavior, engineers gain access to closed-form expressions that explicitly describe how parameters—such as Young's modulus, cross-sectional area, or applied loads—affect the system's response. These expressions enable the direct calculation of partial derivatives of output quantities with respect to input parameters. For example, given a symbolic expression for displacement, one can compute the sensitivity of displacement at a specific point to changes in material stiffness or geometric properties. Similarly, partial derivatives of reactions or internal forces with respect to other parameters can be easily obtained.

For instance, in the third numerical example of Section 6.3, the vertical displacement at Node 2 is calculated symbolically (see Table 11), as shown in Eq. (10):

(10) $$D_{y}=-\frac{P\left(2HL^{2}+\frac{{\left(4H^{2}+L^{2}\right)}^{3/2}}{2}+L^{3}\right)}{4EAH^{2}}$$

Using MATLAB's Symbolic Math Toolbox, partial derivatives of the displacement with respect to parameters such as axial stiffness (*EA*), length (*L*), or height (*H*) can be computed efficiently. Commands such as *diff* for symbolic differentiation and *simplify* for simplifying the resulting expressions make this process straightforward, yielding clear mathematical relationships that highlight the sensitivity of the system's response to design changes. The partial derivates of the displacement *D*_y_ with respect to (*EA*), *L* and *H* can be easily found, as shown in Eqs. (11), (12), (13):

(11) $$\frac{\partial D_{y}}{\partial \left(EA\right)}=-\frac{P\left(2HL^{2}+\frac{{\left(4H^{2}+L^{2}\right)}^{3/2}}{2}+L^{3}\right)}{4{\left(EA\right)}^{2}H^{2}}$$

(12) $$\frac{\partial D_{y}}{\partial L}=-\frac{LP\left(8H+6L+3\sqrt{4H^{2}+L^{2}}\right)}{8EAH^{2}}$$

(13) $$\frac{\partial D_{y}}{\partial H}=\frac{P\left(2HL^{2}+{\left(4H^{2}+L^{2}\right)}^{3/2}-6H^{2}\sqrt{4H^{2}+L^{2}}+2L^{3}\right)}{4EAH^{3}}$$

This symbolic approach provides a distinct advantage over numerical methods, which are typically limited to specific input values and require multiple reruns to evaluate parameter changes. In contrast, symbolic expressions offer general solutions that inherently preserve input-output relationships, enabling effortless and precise sensitivity evaluations without additional computational effort.

This capability is particularly valuable in design optimization, where small parameter adjustments can significantly impact structural performance and cost-effectiveness. By providing closed-form expressions for derivatives, symbolic solutions eliminate the need for approximate numerical differentiation, supporting optimization techniques like Sequential Quadratic Programming (SQP) that rely on accurate derivative information for fast convergence.

For truss design, symbolic sensitivity analysis allows rapid assessment of how variations in geometric or material properties affect structural behavior, facilitating informed design decisions. This clarity and efficiency highlight the symbolic methodology's critical role in advancing structural optimization and bridging the gap between analysis and practical application.

## 9 Balancing complexity and practicality in symbolic computations

The symbolic implementation of MatSA offers flexibility and insight but presents challenges in computational efficiency and scalability, especially for large or complex 2D truss systems. As the number of degrees of freedom, elements, or loading conditions increases, symbolic expressions for stiffness matrices, force vectors, and displacements can become overly large and computationally demanding. Addressing these challenges is critical to ensuring the practical applicability of symbolic MatSA.

Computational time is a critical factor as it increases significantly with the complexity of the truss structure being analyzed. Unlike purely numerical methods, which can solve truss problems in milliseconds on modern computers, symbolic computations involve managing intricate symbolic expressions and performing complex algebraic and matrix operations on them. This additional complexity makes symbolic solutions computationally intensive in comparison.

The second column of Table 22 summarizes the real time required to run each of the five examples 100 times. The third column shows the average time for a single run, obtained by dividing the total time by 100. All computations were performed on a desktop computer running Windows 11 Pro (24H2) with the following specifications: 64 GB RAM, 4 TB NVMe SSD, and a 12th Generation Intel® Core™ i7-12700F processor (running at 2.10 GHz). The table highlights the increasing computational demands of the proposed tool as the complexity of the truss structure grows. While symbolic computations offer significant analytical insights, users should consider this trade-off when handling larger truss systems.

**Table 22:** Computational time needed to run each of the examples∗.

Example #	Time for 100 runs (s)	Average time for a single run (s)
1	52.70	0.53
2	55.41	0.56
3	85.39	0.86
4	136.86	1.37
5	131.41	1.29

Another key challenge in symbolic computation is the exponential growth of expressions as system complexity increases. While simple truss systems produce elegant and manageable symbolic solutions, larger models with many symbolic parameters often result in excessively lengthy and difficult-to-interpret expressions. Such complexity, even when technically valid, reduces the practicality of the symbolic approach. Compact and clear expressions are crucial for generating meaningful insights into structural behavior.

To address this, a hybrid symbolic-numerical approach proves effective and is fully supported by our program. This approach allows certain key parameters to remain symbolic while assigning numerical values to less critical variables. By combining symbolic flexibility with numerical efficiency, the analysis remains scalable and computationally manageable, enabling symbolic MatSA to handle more complex 2D truss systems without excessive computational overhead.

Ultimately, the value of symbolic solutions lies in their clarity and utility. Compact, interpretable solutions enhance understanding and practical application, while excessively complex expressions undermine these benefits. Striking the right balance between symbolic and numerical methods ensures that the advantages of symbolic MatSA are fully realized in 2D truss analysis.

## 10 Conclusions

This study introduces the development and application of an open-source MATLAB program for symbolic Matrix Structural Analysis of 2D trusses subjected to point loads. The freely available source code enables the efficient and precise generation of analytical solutions for plane trusses of any complexity. Beyond its practical engineering applications, the program serves as a valuable educational tool, providing clear and insightful symbolic solutions that enhance understanding of truss behavior.

The program extends its functionality beyond deriving analytical solutions for node displacements, support reactions, and element axial forces. It also facilitates sensitivity analysis by allowing users to compute partial derivatives of output parameters with respect to input variables, using MATLAB's built-in symbolic differentiation commands. This capability is crucial for assessing how changes in design properties, such as material stiffness or geometric dimensions, influence structural response, making the tool invaluable for design exploration and performance evaluation.

The program's capabilities have been demonstrated through various examples, showcasing its potential to deliver precise and insightful analytical solutions for 2D trusses. The results have been rigorously validated using three complementary approaches: comparisons with results from SAP2000 Ultimate (v21.2), EngiLab Truss.2D 2022 Pro (v1.3), and analytical solutions from the literature. These validations demonstrate perfect agreement between the program's outputs and the benchmark results, confirming the accuracy and reliability of the symbolic solutions and affirming the generality of the methodology.

While symbolic solutions offer significant advantages, it is essential to balance complexity and clarity. Although the method is highly scalable for symbolic solutions, solving large truss systems symbolically, it can lead to expressions of significant complexity. This can result in long computation times or memory issues when handling systems with hundreds or thousands of elements. In addition, overly intricate solutions may impede practical application and interpretation. Concise and actionable analytical expressions are preferable as they enhance usability and provide meaningful insights. Efficiency, scalability, and clarity remain key considerations when employing symbolic computations in structural analysis.

A natural extension of this work would be to apply the methodology to other structural systems, such as 3D trusses, where additional complexities like three-dimensional geometry and nodal connectivity can be handled symbolically. This future direction holds great promise for further expanding the applicability of symbolic MatSA and its contributions to structural engineering research and education.

## CRediT authorship contribution statement

**Vagelis Plevris:** Writing – review & editing, Writing – original draft, Visualization, Validation, Supervision, Software, Resources, Project administration, Methodology, Investigation, Formal analysis, Data curation, Conceptualization. **Afaq Ahmad:** Writing – original draft, Visualization, Validation, Software, Methodology.

## Data and code availability statement

No new data were generated for the research described in the article. The source code of the project in MATLAB is available on GitHub at https://github.com/vplevris/SymbolicMatSA-2DTrusses.

## Ethics statement

No experiments involving humans or animals were conducted. The research is purely computational, utilizing symbolic computation methods.

## Declaration of competing interest

The authors declare that they have no known competing financial interests or personal relationships that could have appeared to influence the work reported in this paper.

## Figures and Tables

**Figure:**
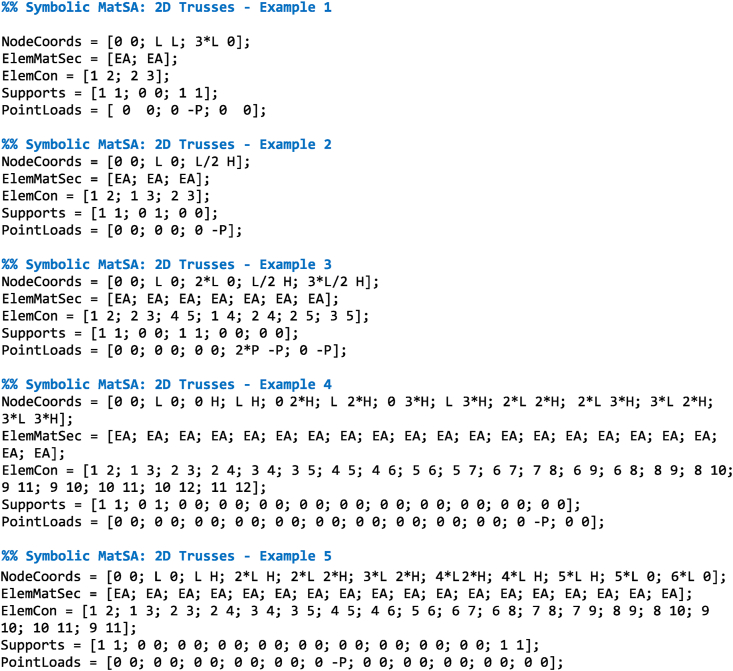


**figs1:**
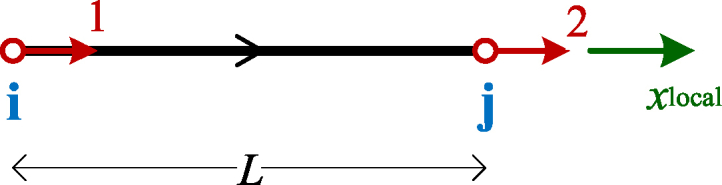


**figs2:**
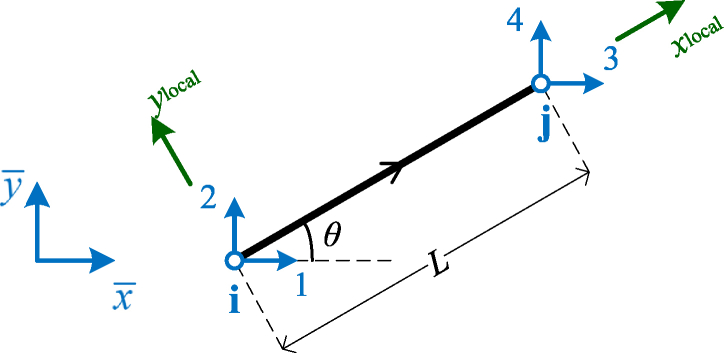


**figs3:**
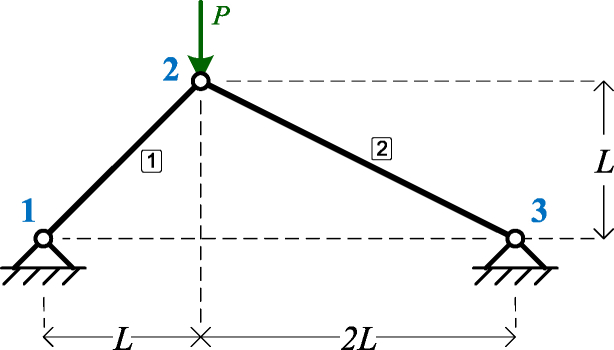


**figs4:**
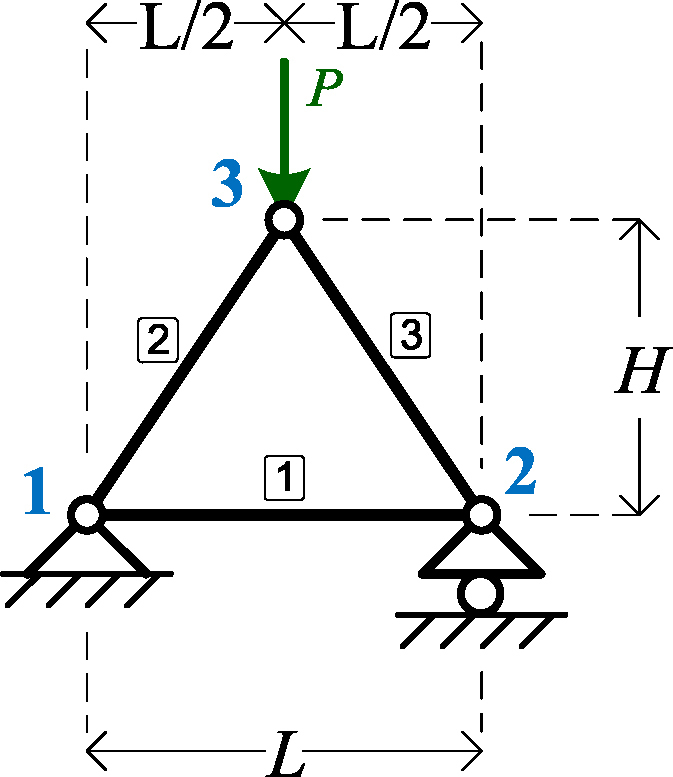


**figs5:**
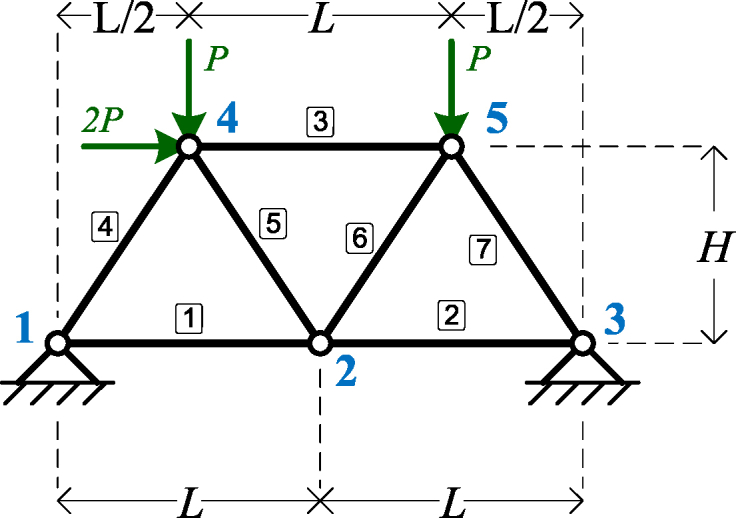


**figs6:**
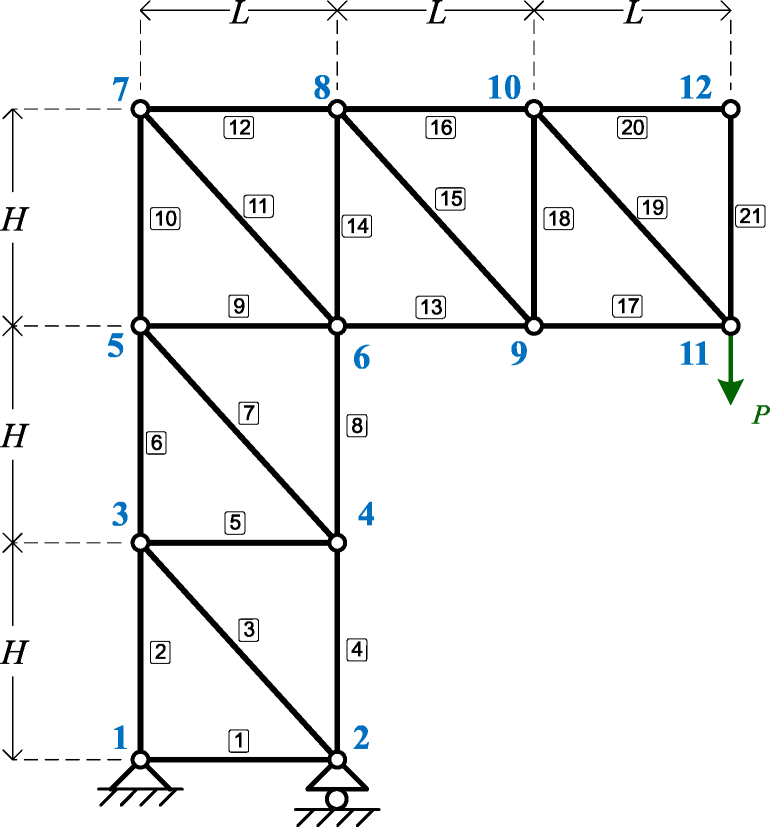


**figs7:**
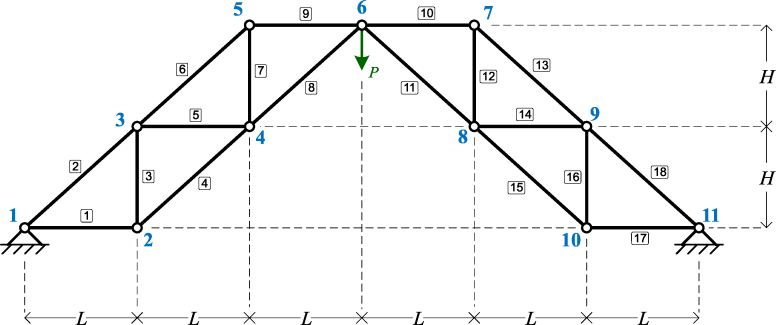


## Appendix

The input files of the five numerical examples are provided in the Appendix.

## References

[1] Liu W.K., Li S., Park H.S. (2022). Eighty years of the finite element method: birth, evolution, and future. Arch. Comput. Methods Eng..

[2] Plevris V., Tsiatas G. (2018). Computational structural engineering: past achievements and future challenges. Frontiers in Built Environment.

[3] Mazumder S., Mazumder S. (2016). Numerical Methods for Partial Differential Equations.

[4] Plevris, V. and A. Ahmad, Deriving Analytical Solutions Using Symbolic Matrix Structural Analysis: Part 1 - Continuous Beams*.* ArXiv e-prints, 2024(arXiv:2411.03514) DOI: 10.48550/arXiv.2411.03514. PMC12059071 40335595

[5] Papadrakakis M., Lagaros N.D., Tsompanakis Y., Plevris V. (2001). Large scale structural optimization: computational methods and optimization algorithms. Arch. Comput. Methods Eng..

[6] Lagaros N.D., Plevris V., Kallioras N.A. (2022). The mosaic of metaheuristic algorithms in structural optimization. Arch. Comput. Methods Eng..

[7] Moses J. (2012). Macsyma: a personal history. J. Symbolic Comput..

[8] Farahmand-Tabar S., Aghani K., Farahmand-Tabar S., Aghani K. (2024). Practical Programming of Finite Element Procedures for Solids and Structures with MATLAB®.

[9] Geisel A., Svaricek F. (2019). A MATLAB toolbox for structural analysis of linear systems. IFAC-PapersOnLine.

[10] Yang B., Yang B. (2023). Stress, Strain, and Structural Dynamics.

[11] Kattan P.I. (2008).

[12] Papazafeiropoulos G., Plevris V. (2024). OpenSeismoMatlab: new features, verification and charting future endeavors. Buildings.

[13] Yang B., Yang B. (2023). Stress, Strain, and Structural Dynamics.

[14] Yang B., Yang B. (2023). Stress, Strain, and Structural Dynamics.

[15] François S. (2021). Stabil: an educational Matlab toolbox for static and dynamic structural analysis. Comput. Appl. Eng. Educ..

[16] Plevris V., Solorzano G. (2022). A collection of 30 multidimensional functions for global optimization benchmarking. Data.

[17] Andreassen E., Clausen A., Schevenels M., Lazarov B.S., Sigmund O. (2011). Efficient topology optimization in MATLAB using 88 lines of code. Struct. Multidiscip. Optim..

[18] Sigmund O. (2001). A 99 line topology optimization code written in Matlab. Struct. Multidiscip. Optim..

[19] Solorzano G., Plevris V. (2020). Optimum design of RC footings with genetic algorithms according to ACI 318-19. Buildings.

[20] Arora J.S., Arora J.S. (2012). Introduction to Optimum Design.

[21] Ferreira A.J.M., Fantuzzi N. (2020). MATLAB Codes for Finite Element Analysis: Solids and Structures.

[22] MathWorks (2024). Symbolic Math Toolbox™ user's guide. https://www.mathworks.com/help/pdf_doc/symbolic/symbolic_ug.pdf.

[23] Lynch S. (2004). Dynamical Systems with Applications Using MATLAB®.

[24] Attaway S., Attaway S. (2019). MATLAB.

[25] Valentine D.T., Hahn B.D., Valentine D.T., Hahn B.D. (2023). Essential MATLAB for Engineers and Scientists.

[26] Asadi F., Asadi F. (2023). Applied Numerical Analysis with MATLAB®/Simulink®: for Engineers and Scientists.

[27] Mureşan M. (2017).

[28] Smith E. (2022). Introduction to the Tools of Scientific Computing.

[29] Cywiak M., Cywiak D. (2021). Multi-Platform Graphics Programming with Kivy: Basic Analytical Programming for 2D, 3D, and Stereoscopic Design.

[30] Plevris, V. and A. Ahmad, Deriving Analytical Solutions Using Symbolic Matrix Structural Analysis: Part 2 - Plane Trusses. ArXiv e-prints, 2024(arXiv:2411.16573) DOI: 10.48550/arXiv.2411.16573. PMC11874738 40034312

[31] Parisi F., Ruggieri S., Lovreglio R., Fanti M.P., Uva G. (2024). On the use of mechanics-informed models to structural engineering systems: application of graph neural networks for structural analysis. Structures.

[32] Eriksson A., Pacoste C. (1999). Symbolic software tools in the development of finite elements. Comput. Struct..

[33] Amberg G., Tönhardt R., Winkler C. (1999). Finite element simulations using symbolic computing. Math. Comput. Simulat..

[34] Pavlović M.N. (2003). Symbolic computation in structural engineering. Comput. Struct..

[35] Levy R., Chen C.-S., Lin C.-W., Yang Y.-B. (2004). Geometric stiffness of membranes using symbolic algebra. Eng. Struct..

[36] Murphey T. (2006). 47th AIAA/ASME/ASCE/AHS/ASC Structures, Structural Dynamics, and Materials Conference.

[37] Renton J.D. (1995). Automated derivation of structural formulae. Comput. Struct..

[38] Skrinar M., Pliberšek T. (2012). On the derivation of symbolic form of stiffness matrix and load vector of a beam with an arbitrary number of transverse cracks. Comput. Mater. Sci..

[39] Roque C.M.C. (2014). Symbolic and numerical analysis of plates in bending using Matlab. J. Symbolic Comput..

[40] Tinkov D.V. (2015). Comparative analysis of analytical solutions to the problem of truss structure deflection. Magazine of Civil Engineering.

[41] Kirsanov M., Tinkov D. (2018).

[42] Kirsanov M. (2018). Analytical calculation of the frame with an arbitrary number of panels. Magazine of Civil Engineering.

[43] Dasgupta G., Dasgupta G. (2018). Finite Element Concepts: A Closed-form Algebraic Development.

[44] Öchsner A., Makvandi R., Öchsner A., Makvandi R. (2019). Finite Elements for Truss and Frame Structures: an Introduction Based on the Computer Algebra System Maxima.

[45] Öchsner A., Makvandi R., Öchsner A., Makvandi R. (2019). Finite Elements for Truss and Frame Structures: an Introduction Based on the Computer Algebra System Maxima.

[46] Mashru N., Tejani G.G., Patel P., Khishe M. (2024). Optimal truss design with MOHO: a multi-objective optimization perspective. PLoS One.

[47] Kumar S. (2025). Optimization of truss structures using multi-objective cheetah optimizer. Mech. Base. Des. Struct. Mach..

[48] Kumar S. (2023). A two-archive multi-objective multi-verse optimizer for truss design. Knowl. Base Syst..

[49] Charalampakis A.E., Chatzigiannelis I. (2018). Analytical solutions for the minimum weight design of trusses by cylindrical algebraic decomposition. Arch. Appl. Mech..

[50] Muftu S., Muftu S. (2022). Finite Element Method.

